# Investigation of *CTX‐M* Type Extended‐Spectrum β‐Lactamase, Carbapenem and Colistin Resistance in Enterobacterales Isolated From Dairy Cattle in Turkey

**DOI:** 10.1002/vms3.70523

**Published:** 2025-07-24

**Authors:** Metin Yalcin, Sinem Özlem Enginler, Mert Ahmet Kuşkucu, Mert Sarılar, Selma Yalçın, Ömer Küçükbasmacı

**Affiliations:** ^1^ Department of Medical Microbiology Cerrahpasa Faculty of Medicine Istanbul University‐Cerrahpasa Istanbul Turkey; ^2^ Faculty of Veterinary Medicine Istanbul University‐Cerrahpasa Istanbul Turkey; ^3^ Department of Medical Microbiology Koç University Istanbul Turkey

**Keywords:** antibiotic resistance | carbapenemase | colistine | dairy cattle | extended‐spectrum β‐lactamase (ESBL)

## Abstract

**Background:**

The increasing prevalence of antimicrobial resistance in animals, particularly the spread of multidrug‐resistant Enterobacterales, poses a significant zoonotic and public health risk.

**Objective:**

The aim of this study was to investigate extended‐spectrum β‐lactamase (ESBL), carbapenem and colistin resistance among Enterobacterales in faecal swabs of dairy cattle.

**Methods:**

A total of 400 samples were cultured on Mac Conkey screening media for ESBL, carbapenem and colistin resistance. The grown Enterobacterales were identified by MALDI‐TOF‐MS, followed by ceftriaxone, cefotaxime and ceftazidime resistance and double disk synergy. ESBL resistance genes were identified by polymerase chain reaction (PCR) and Sanger sequencing. Bacteria grown on colistin screening media were investigated for colistin resistance by EUCAST microbroth dilution method.

**Results:**

A total of 89 (22.25%) of the bacteria grown from 400 samples were identified as potential ESBL‐producing Enterobacterales members. A number of 53 (59.5%) of them were identified as ESBL *blaCTX‐M* as a result of PCR, and 10 of them were identified as *blaCTX‐M‐15/28/36/66* as a result of sequencing. None of the samples cultured on carbapenem medium grew. A total of 18 samples grown in colistin medium were found to be colistin sensitive by broth microdilution. Genotypes were not included in the study. All isolated bacteria were identified as *Escherichia coli*.

**Solution:**

In this study, *blaCTX‐M‐15* and its derivatives, which are common in humans, were also found to be the predominant ESBL type in animals. Monitoring resistance in animals together with resistance in human infections may provide more important data on the spread of resistance.

## Graphical abstract

This study reveals the presence of *blaCTX‐M*‐type ESBL‐producing *E. coli* in dairy cattle in Turkey, highlighting the zoonotic potential of antimicrobial resistance. No carbapenem or colistin resistance was detected.

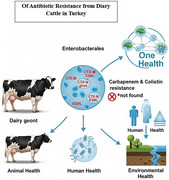

## 1 Introduction

Antimicrobial resistance (AMR) is a serious global threat to both human and animal health. Resistant bacteria relevant to human health can emerge in farm animals; therefore, active surveillance in food‐producing animals is also essential. The impact of AMR observed in animals used for food production on public health is being addressed globally through the One Health approach (Lin et al. 2022). The European Food Safety Association (EFSA) has recognized foodborne AMR as a ‘biohazard’ (EFSA 2012).

Members of Enterobacterales are widely abundant in nature. They are responsible for 80% of the diseases caused by Gram‐negative rods such as gastroenteritis, urinary tract infection, pneumonia, meningitis, endotoxic shock and sepsis (Murray et al. 2016).

β‐Lactam group antibiotics are the most widely used antibiotic group due to their high selective toxicity, broad spectrum, low side effects, use in all age groups and high distribution in body fluids. β‐Lactam group antibiotics are also widely used in the treatment of animal infections (Dağlar and Öngüt 2012). Colistin is an antimicrobial agent effective against multidrug‐resistant Gram‐negative bacteria (Donnenberg 2016). In 2016, the World Health Organization (WHO) classified colistin as a critically important drug for microbial therapy (WHO 2016).

Carbapenem‐resistant Enterobacterales (CRE) are a group of Gram‐negative bacteria that have become resistant to carbapenems, a class of antibiotics that are last‐line therapy for many serious infections (Jin et al. 2018). CRE can be found in a variety of settings, including hospitals, nursing homes and other healthcare facilities. However, it is increasingly becoming more abundant in environments and among recently hospitalized or non‐hospitalized people and farm animals (Tuhamize and Bazira 2024). The emergence of CRE in these various settings has raised concerns about the rapid spread of these multidrug‐resistant pathogens (Zaidi et al. 2023).

The one‐medicine approach is of particular importance for microbiology as a unifying discipline linking health between humans, animals and the environment. Given that more than 60% of infectious diseases are caused by the transmission of an infectious agent from animals, the use of a systematic single‐medicine approach has great potential to reduce global health threats from infectious diseases (Atlas et al. 2010).

Within this scope of single‐medicine approach, in our study, we aimed to investigate extended‐spectrum β‐lactamase (ESBL), carbapenem and colistin‐resistant bacteria belonging to the Enterobacterales order in the faecal microbiota of cattle, which pose a threat to public health, and to determine phenotypic and genotypic resistance profiles for the resistant strains. We think that our study will provide an important cross section of the current resistance situation in farm animals by evaluating ESBL carbapenem and colistin resistance analysis.

## 2 Materials and Methods

### 2.1 Collection of Samples

Samples were collected from various farms in Istanbul and Izmir. A total of 400 samples were included in the study. Samples taken from faecal swabs of fattening animals were placed in Carry‐Blair transport medium. They were kept at 2–8°C and studied within 24 h (Nagata et al. 2019).

A total of 400 lactating cows from various commercial dairy farms located in the Marmara Region of Türkiye were included in the study. All animals enrolled in the study were of the Holstein‐Friesian breed and were raised under standard commercial dairy farming conditions, including regular milking and routine veterinary supervision. The cows were in mid‐lactation and ranged in age from 3 to 8 years. At the time of the study, all animals were clinically healthy and actively involved in commercial milk production. According to farm records, none of the animals had received antibiotic treatment within the 3 months prior to the study, minimizing the potential influence of recent antimicrobial exposure on the results.

### 2.2 Culture Preparation and Determination of Phenotypic Resistance

Samples were incubated in Mac Conkey Agar (Biolab, Hungary) screening medium containing 1 µg/mL ceftriaxone and Mac Conkey Agar screening medium containing 2 µg/mL colistin sulphate at 37°C for 18–24 h. After incubation, colonies grown on Mac Conkey Agar screening medium containing 1 µg/mL ceftriaxone were identified as potential producing Enterobacterales members, whereas bacteria grown on Mac Conkey screening medium containing 2 µg/mL colistin were identified as potential colistin‐resistant Enterobacterales members (Sow et al. 2024; Anyanwu et al. 2021; M'Zali et al. 2000).

All isolated bacteria were identified by MALDI‐TOF MS (Bruker, Germany).

For phenotypic determination of ESBL resistance, the following steps were followed: Double Disk Synergy method was performed on Mueller Hinton agar (HiMedia, India) medium using Ceftriaxone (30 µg/mL Bioanalyse, TR), Ceftazidime (30 µg/mL Bioanalyse, TR), Cefotaxime (30 µg/mL Bioanalyse, TR) and Amoxicillin–Clavulanic acid (30 µg/mL Bioanalyse, TR) disks (M'Zali et al. 2000). After incubation at 37°C for 18–24 h, resistant strains were determined according to EUCAST standards (EUCAST 2024).

For phenotypic determination of colistin resistance, the following steps were followed: Colonies grown on colistin screening medium were studied as potential colistin‐resistant colonies by broth microdilution method according to EUCAST standards. In addition, antibiotic susceptibility test was performed by Kirby–Bauer disk diffusion method using colistin (10 µg/mL Bioanalyse, TR) disks.

It was planned to confirm the carbapenem resistance of Enterobacterales member bacteria grown in the carbapenem screening test with meropenem (30 µg/mL Bioanalyse, TR) disk and to carbapenemase by CIM (Carbapenem Inactivation Method) and genes by polymerase chain reaction (PCR) method when found positive.

All strains were transferred to 1 mL Eppendorf tubes containing 10% glycerol tryptic soy broth (HiMedia, India) and stored at −20°C.

### 2.3 Determination of Genotypic Resistance

Stock strains were removed from −20°C and brought to room temperature. They were inoculated on Mueller Hinton agar medium and incubated at 37°C for 18–24 h. The strains were inoculated on biochemical identification media and chromogenic agar (Chromagar UTI HiMedia, India) to confirm that stocks do not contain any contamination.

ESBL‐resistant *Escherichia coli* strains were analysed by PCR to detect *blaCTX‐M*‐encoding genes. *BlaCTX‐M* resistance gene was studied because it is the most common, virulent and highly antibiotic‐resistant group (D'Andrea et al. 2013; Clermont et al. 2008). 16s rRNA was also used as an internal control to exclude false negative results due to PCR inhibition, and negative controls were included in each PCR setup to exclude false positive results due to cross‐contamination. Amplifications were performed using primers listed in Table 1 (Chen et al. 2004; Kuskucu et al. 2016).

**TABLE 1:** Polymerase chain reaction (PCR) primers.

Primers	Forward primer (5′‐3′)	Reverse primer (5′‐3′)	Size	*T*_m_ (°C)	GenBank
*BlaCTX‐M*	ATCTGACGCTGGGTAAAGC	ATATCGTTGGTGGTGCCATA	162	50	JQ686201

### 2.4 DNA Sequence Analysis

Sanger sequence analysis was performed among selected ESBL‐resistant bacteria. Sanger Sequence Analysis: For *blaCTX‐M* gene region research; PCR products were purified with ExoSAP‐IT (Thermo Scientific, USA) as the first step. In the second step, ‘Big Dye reaction mix’ (Thermo Scientific, USA) was used for ‘cycle sequencing’. Finally, column formation and purification were performed with Sephadex (Sigma Aldrich, St. Louis, Missouri, United States). The purified PCR products were loaded into a genetic analyser (ABI 3130GA). The results were compared with NCBI. A total of 10 *blaCTX‐M* isolates with similar resistance profiles were selected from each farm and sequenced.

## 3 Results

Of the 400 samples grown on screening medium, 89 (22.25%) were identified as potentially ESBL‐resistant Enterobacterales. Of the 400 samples cultured on colistin screening medium, 18 (4.5%) were identified as potentially colistin resistant. None of the samples cultured on carbapenem screening medium grew.

All bacteria grown on ESBL and colistin screening media were identified as *E. coli*.

Antibiotic susceptibilities of ESBL‐suspected strains were determined by Kirby–Bauer disk diffusion method, and ESBL production was phenotypically confirmed by double disk synergy method. All strains were resistant to Ceftriaxone, Ceftazidime and Cefotaxime.

Phenotypic colistin confirmation was performed by broth microdilution method on 18 potentially colistin‐resistant *E. coli* strains isolated. All strains were found to be susceptible below colistin MIC ≤ 2 µg/mL. Kirby–Bauer disk diffusion method revealed that all isolates had colistin zone diameter >15 mm. As all isolates were found to be colistin sensitive, they were not included in the genotypic study.

### 3.1 ESBL Genotyping Results

β‐Lactamase genes carried by the 89 strains found to produce ESBL *blaCTX‐M* were analysed. It was observed that 53 (59.5%) of the samples carried the *blaCTXM* gene. The ESBL CTX‐M gene ratio is given in Table 2. ESBL CTX‐M gen subgroups by farm are given table (Table 2, 3 and 4).

**TABLE 2:** Extended‐spectrum β‐lactamase (ESBL) types determined by polymerase chain reaction (PCR).

β‐Lactamase type	*n*	%
*blaCTXM*	53 pieces	59.5

**TABLE 3:** Distribution of extended‐spectrum β‐lactamase (ESBL) positivity according to farms.

Farm	Number	Positive	Negative	*blaCTX‐M*
1. İstanbul	70	16 (22.8%)	54 (77.2%)	10 (62.5%)
2. İstanbul	30	5 (16.6%)	25 (83.4%)	3 (60%)
3. İstanbul	120	26 (21.6%)	94 (78.4%)	16 (61.5%)
4. İzmir	180	42 (23.3%)	138 (76.7%)	24 (57.14%)

**TABLE 4:** Extended‐spectrum β‐lactamase (ESBL) gene distribution rates according to farms as a result of sequence.

Farm	*blaCTX‐M‐15*	*blaCTX‐M‐28*	*blaCTX‐M‐36*	*blaCTX‐M‐66*
1. *n* = 13	—	1 (7.6%)	1 (7.6%)	—
2. *n* = 5	—	1 (20%)	—	1 (20%)
3. *n* = 26	1 (3.8%)	—	1 (3.8%)	—
4. *n* = 42	1 (2.3%)	2 (4.7%)	—	1 (2.3%)

### 3.2 DNA Sequence Analysis Result

ESBL *blaCTX‐M* resistance genes were identified by Sanger sequencing of 10 selected *blaCTX‐M* positive samples. Accordingly, two of these genes were identified as *blaCTX‐M‐15*, 4 *as blaCTX‐M‐28*, 3 as *blaCTX‐M‐36* and 1 as *blaCTXM‐66*. All identified resistance genes are in *blaCTX‐M* group 1.

## 4 Discussion

In our study, ESBL‐resistant *E. coli* was isolated from 89 (22.25%) of 400 faecal swabs collected from fattening animals. Of the isolated strains, 53 (59.5%) had *blaCTX‐M* gene. DNA sequence analysis of 10 selected *blaCTX‐M* *strains* revealed that *2 of them had blaCTX‐M‐15*, *4 had blaCTX‐M‐28*, 2 had *blaCTX‐M‐36*, *and 2 had blaCTX‐M‐66* gene type. All identified resistance genes were in *blaCTX‐M* group 1. The clinically important *blaCTX‐M‐15* origin was detected in both Marmara and Aegean regions.

In addition, no colistin‐ and carbapenem‐resistant Enterobacterales member bacteria were found in any of the 400 swab samples.

When the studies conducted were examined, it was observed that the rates of ESBL bacteria in livestock increased every year. Especially *blaCTX‐M* gene has been observed to spread rapidly in the world (Doerr et al. 2023).

In our study, *E. coli* was isolated from the faeces of dairy cattle. *E. coli* is an important Gram‐negative bacterium that typically resides harmlessly in the intestinal tract of animals; however, it has the capacity to rapidly acquire antibiotic resistance through horizontal gene transfer. This ability contributes to the emergence of multidrug‐resistant strains, which significantly complicates infection treatment and clinical management (Narwal et al. 2025).

According to the WHO Antimicrobial Consumption (AMC) Network Working Group, Turkey ranks first among OECD (Organisation for Economic Co‐operation and Development) countries in terms of human antibiotic consumption, with 35.7 DID (Defined Daily Dose per 1000 inhabitants per day) (T:C sağlık bakanlığı 2024). Although data on antibiotic use in food‐producing animals in Turkey are quite limited, the country ranks among the top in Europe with a reported usage of 69 mg/PCU (Population Correction Unit). In comparison, this figure is 69.9 mg/PCU in Germany, 25.7 mg/PCU in the United Kingdom and 2.1 mg/PCU in Norway (European Medicines Agency 2022).

In a study conducted in China in 2020 in bovine mastitis, *blaCTX‐M‐28* and *blaCTX‐M‐66* genes were isolated (Shafiq et al. 2021). In addition, in a study conducted in China in 2012, *blaCTX‐M‐28* and *blaCTX‐M‐36* genes were isolated in *E. coli* of human and swine origin. These studies are similar to our study (Tian et al. 2012).

According to a study conducted in Bavaria, Germany in 2022, 27 (22.5%) of 120 faecal samples from cattle on a farm were found to be ESBL resistant. It was reported that 26 (96.2%) of the ESBL‐resistant strains had the *blaCTX‐M* *(CTX‐M‐1* and *CTX‐M‐15*) gene. It was observed that this study had a very similar resistance rate with our study (Homeier‐Bachmann et al. 2022).

In a comprehensive study conducted in Canada, France and Germany in 2019 from various host types (clinical human, healthy and sick animals, meat and wastewater), *blaCTX‐M* resistance genes were found to be *blaCTX‐M‐1/14/15* genes. Although the differences with the *blaCTX‐M* gene types in our study are explained by geographical differences, the similarities in *blaCTX‐M* gene types are also similar to our study (Zamudio et al. 2024).

According to a multicentre surveillance study conducted in Europe in 2021, 31% of 99 (3.3%) ESBL‐resistant *E. coli* strains isolated from 2993 samples of cattle, chicken and pig faeces in various slaughterhouses were found to have the *blaCTX‐M* (CTX‐M‐1/‐2/‐14/‐15 gene. When compared with our study, it was observed that the rate of *blaCTX‐M* gene was quite similar. The spread of the *blaCTX‐M‐15* gene was similar. The differences in ESBL resistance gene types and rates can be explained by geographical location and clonal spread (Ewers et al. 2021).

Studies on ESBL types and prevalence in bovine livestock are limited in our country. Our team was the first to study ESBL resistance in livestock in the veterinary field in our country (Kucukbasmacı 2008). In our 2008 study, *blaTEM* and *bla SHV* were prevalent, whereas in our current study, *blaCTX‐M* was prevalent.

In a study conducted by Yıldırım D, Pehlivanoğlu F. on 150 calves in Burdur in 2016, 44 (29.1%) *E. coli* samples were isolated. All of the isolated strains were identified as *blaCTX‐M* (group 1) (Pehlivanoglu 2019). The high ESBL resistance rate and *blaCTX‐M* group 1 strains are quite similar to our study.

Aslantaş et al., in their study conducted in Hatay province, isolated 26 (8.3%) ESBL‐forming *E. coli* strains from faecal samples of 312 cattle fattening animals. The *blaCTXM (CTX‐M‐1/‐3/‐15)* resistance gene was found in 13 (50%) of the strains (Aslantaş et al. 2017). The high rate of *blaCTX‐M* gene and the excess of *blaCTX‐M‐15* and *blaCTX‐M* group 1 are in parallel with our study. Other differences in *blaCTX‐M* gene type are thought to be due to both regional differences and differences in the molecular method used.

The presence of carbapenemases in animals raises concerns about the possibility of transmission of antibiotic‐resistant bacteria to humans. If these bacteria can pass from animals to humans, they pose a significant risk to public health by reducing the effectiveness of treatment for serious infections (Tuhamize and Bazira 2024).

CRE member bacteria have been reported in livestock in many countries. In Canada, the presence of carbapenem‐resistant *E. coli* was reported in a study conducted on livestock in 2023 (Zaidi et al. 2023). In a study conducted in Spain, carbapenemase gene was detected in dairy cattle (Zaidi et al. 2023).

CRE member bacteria are not common in livestock in our country for now. Babacan (2023) detected the first carbapenem resistance in *E. coli* in cattle with mastitis in 2023 in Balıkesir.

In our study, no CRE were found in carbapenem screening. Considering the spread of carbapenem resistance, we think that more farms and livestock should be screened with more budget.

The mcr‐1 gene (mobile colistin resistance gene‐1), which causes the rapid spread of colistin resistance and is carried on a plasmid, was first reported in China in 2015 (Liu et al. 2016). Subsequently, the colistin resistance gene mcr has been identified in various parts of the world such as Denmark, the United States, Brazil, the Netherlands and Egypt. Today, 10 types of mcr genes have been identified (mcr‐10) (Valiakos and Kapna 2021).

According to a study conducted in China in 2022, colistin resistance was studied in *E. coli* strains isolated from chicken, pig, cattle and dog faeces. As a result, 35 (51.47%) of the strains isolated from 68 cattle faeces were found to be colistin‐resistant *E. coli*. As colistin resistance is spread through plasmids, it can be said that clonal spread causes such high colistin resistance. This may also explain the low colistin resistance rate in livestock in our country (Li et al. 2022).

According to a multicentre surveillance study conducted in Europe in 2021, 1 colistin‐resistant *E. coli* strain was detected in 1 of 2993 samples of cattle, chicken and pig faeces in various slaughterhouses. In this study, in which many European countries participated and the number of samples was quite high, the detection of 1 *E. coli* strain with mcr‐1 colistin resistance gene indicates that the plasmid‐mediated mcr resistance gene has not yet spread in livestock in these regions (Liu et al. 2016).

According to a study published in the Czech Republic in 2025, ESBL genes were detected in 43.8% of *E. coli* strains isolated from rectal swabs of dairy cattle and their caretakers. These genes were identified as *blaCTX‐M‐1/8/14/15* genotypes. Additionally, the colistin resistance gene *mcr‐1* was detected in one isolate. The ESBL resistance rate reported in this study is highly comparable to that observed in our study. The variation in ESBL gene types may be attributed to geographical differences. Similarly, the low number of isolates carrying the colistin resistance gene is consistent with our findings (Masarikova et al. 2025).

According to a study conducted in China in 2019, ESBL resistance genes were detected in 20% of 249 *E. coli* strains isolated from 2038 mastitic milk samples. Additionally, the colistin resistance gene *mcr‐1* was identified in five isolates. Notably, all five *mcr‐1*‐positive isolates also carried the ESBL gene *blaCTX‐M‐15*. When compared with our study, the prevalence and types of ESBL resistance genes show considerable similarity. The co‐occurrence of both colistin and ESBL resistance genes in the same isolates aligns with the public health concerns highlighted in our study (Liu et al. 2020).

In a study published in France in 2022, more than 3% of isolates obtained from 397 faecal samples collected from chickens, goats, cattle and pigs harboured the acquired colistin resistance genes *mcr‐1* and *mcr‐3*. From the perspective of the One Health approach, the detection of colistin resistance genes at a relatively high frequency in various farm animals underscores the importance of colistin resistance gene screening, as conducted in our study (Hamame et al. 2022).

Another study from China in 2022 reported the presence of the colistin resistance gene *mcr‐1* in 7 out of 79 *E. coli* isolates obtained from cats, dogs and environmental samples (including faeces, wounds and surfaces). These *mcr‐1*‐positive isolates also carried the ESBL gene *blaCTX‐M‐65*. The coexistence of multiple resistance genes in *E. coli* strains, along with the potential for plasmid‐mediated gene transfer, may accelerate the emergence of multidrug‐resistant bacteria and facilitate transmission from companion animals to humans. This highlights the significance of our study within the framework of the One Health approach (Lin et al. 2022).

The first mcr gene in our country was reported in a study by Kürekçi et al. All of the strains isolated from 80 chicken meat taken from Hatay and Adana regions were identified as *E. coli*, and 4 of them (5%) were reported to carry mcr‐1 colistin resistance gene (Kurekci et al. 2018). In a study conducted in 2021 in the vicinity of our study area, colistin resistance was detected in 7.5% of 200 *E. coli* isolated from chicken faeces, but no mcr gene was detected (Erzaim and Ikiz 2021). Although colistin is frequently used in intensive care units in our centre, no mcr gene was found in the previous study by Borsa et al. (2019). The fact that colistin‐resistant strains were not phenotypically detected in our study is a promising situation in terms of the spread of colistin resistance, and this situation should be maintained with the necessary controls and monitoring.

In our study, the clinically important *blaCTX‐M* (*blaCTX‐M‐15*, *blaCTX‐M‐28*, *blaCTX‐M‐36*, *blaCTXM‐66*) gene was commonly found among ESBL‐producing strains. These genes were obtained from *E. coli*, which are frequently isolated from humans, the environment and animals. In our country and in the world, *blaCTX‐M‐15* is the most common origin encountered in clinical trials (Silva‐Sánchez et al. 2024). Bla*CTX‐M‐15* gene is located in *blaCTX‐M* group 1 together with *blaCTX‐M‐28/36/66* genes, and these genes are separated from each other due to one or a few amino acid differences (D'Andrea et al. 2013; Rossolini et al. 2008). These genes are 95% similar. Especially *blaCTX‐M‐28* differs from *blaCTX‐M‐15* by a single amino acid difference (Alfaresi et al. 2018). We think that this difference may be due to the type of antibiotic used, the biochemical activity of the bacteria or host or changes in environmental conditions. As the primers used in our study were able to detect differentiation in the *blaCTX‐M* gene, they show the diversification within *CTX‐M* group 1 in more detail.

## 5 Conclusion

Our study showed that *bla‐CTX‐15*, which is common in humans, is also the main ESBL type in animals in Istanbul and Izmir regions. Although colistin resistance has been reported in many studies conducted in Türkiye, variations in detection methods and interpretation criteria may lead to inconsistencies in the reported resistance levels. Standardized phenotypic and genotypic approaches are essential for obtaining accurate and reliable results.

Thanks to our study, the spread of the clinically important *blaCTX‐M‐15* origin in animals in both Marmara and Aegean regions has been reported. Our study also showed the prevalence of *blaCTX‐M‐28/36/66* genes in our country. Considering the single‐medicine approach, it is an important cross section in terms of clinical, environmental and veterinary microbiology. We think that our study will support the efforts to prevent the spread of antibiotic resistance in all these branches. These data suggest that further molecular methods and evaluation with a larger sample are required and that the establishment of a country‐based monitoring system may be effective in this regard.

## Author Contributions

**Metin Yalcin**: conceptualization, visualization, writing – original draft, writing – review and editing, methodology. **Sinem Özlem Enginler**: conceptualization, writing – original draft, methodology. **Mert Ahmet Kuşkucu**: conceptualization, formal analysis, investigation, software. **Mert Sarılar**: conceptualization, data curation, investigation. **Selma Yalçın**: conceptualization, investigation. **Ömer Küçükbasmacı**: conceptualization, data curation, writing – review and editing, writing – original draft, project administration.

## Ethics Statement

All samples were obtained after informed consent and with the approval of the Istanbul‐Cerrahpasa University Faculty of Medicine Ethics Committee: E‐83045809‐604.01.02‐3037, date: 07.01.2021. All experiments within the study were carried out in compliance with the relevant laws and guidelines, under the ethical standards of the Declaration of Helsinki.

## Conflicts of Interest

The authors declare no conflicts of interest.

## Peer Review

The peer review history for this article is available at https://publons.com/publon/10.1002/vms3.70523.

## Data Availability

The datasets generated during and/or analysed during the current study are available from the corresponding author upon reasonable request.
